# Repeated-sprint training with low lung volume voluntary hypoventilation performed continuously throughout each set in healthy females

**DOI:** 10.3389/fphys.2025.1713797

**Published:** 2026-01-15

**Authors:** Léa Devantay, Grégoire P. Millet, Antoine Raberin

**Affiliations:** Institute of Sports Sciences, University of Lausanne, Lausanne, Switzerland

**Keywords:** cycling, females, hypoxia, repeated sprints, voluntary hypoventilation at low lung volume

## Abstract

**Purpose:**

This study aimed to investigate the acute responses induced by a session of repeated-sprint training in hypoxia (RSH) induced by voluntary hypoventilation at low lung volume (VHL) performed continuously throughout the exercise in healthy females.

**Methods:**

Thirteen females performed, in a randomized order, two sessions of repeated sprints (three sets of eight 10-s all-out sprints): with normal breathing (RSN) vs. with VHL performed continuously throughout each set (RSH-VHL). Peak and mean power output, heart rate, stroke volume, cardiac output, pulse oxygen saturation, muscle oxygenation in the vastus lateralis and the biceps brachii, blood lactate concentration, rate of perceived exertion and perceived difficulty of breathing and pedalling were assessed.

**Results:**

RSH-VHL did not induce desaturation (97.5 ± 2.0 for RSH-VHL vs. 98.0% ± 1.6% for RSN; p = 0.243) nor greater muscle deoxygenation in the vastus lateralis (mean minimum tissue saturation index: 62.3% ± 4.3% vs. 61.5% ± 4.4%; p = 0.193) or the short head of the biceps (36.6% ± 10.0% vs. 34.2% ± 13.7%; p = 0.320). Significantly lower training load indices were observed from the first set onwards during RSH-VHL compared with RSN: mean peak power output (311 ± 45 vs. 382 ± 46 W; p < 0.001) and blood lactate concentration (6.8 ± 2.9 vs. 9.9 ± 3.0 mmol/L; p = 0.003). The perceived difficulty of breathing was higher during RSH-VHL than RSN from the first set onwards (8.2 ± 2.2 vs. 6.0 ± 0.9; p = 0.022).

**Conclusion:**

This study showed that, although participants reported increased breathing difficulty during RSH-VHL performed continuously, this condition did not result in significant systemic or local hypoxia. Moreover, it led to a lower training load compared to RSN. When VHL is performed continuously throughout each set, rather than only during sprints, it may be too strenuous, inducing a significant reduction in training load.

## Introduction

Repeated-sprint training in hypoxia (RSH) have been shown to improve repeated sprint ability (RSA) to a greater extent than repeated-sprint training in normoxia (RSN); e.g., they lead to greater performance and power output across sprints, associated with improved fatigue resistance (Brocherie et al., 2017; Faiss et al., 2024; Millet et al., 2019). Moreover, a recent study by Piperi et al. (2024) demonstrated that RSH improved performance during repeated sprints in females similarly to males.

Unlike RSH, which requires access to a hypoxic environment, repeated-sprint training in hypoxia induced by voluntary hypoventilation at low lung volume (RSH-VHL) can be performed without specific equipment. Indeed, VHL induces a hypoxic stimulus sufficient to cause severe oxygen (O_2_) desaturation (Millet et al., 2019; Woorons et al., 2007; Woorons et al., 2017). It is well established that pulse oxygen saturation (SpO_2_) is significantly lower during an RSH-VHL session compared to RSN (Ait Ali Braham et al., 2024; Fornasier-Santos et al., 2018; Woorons et al., 2016; Woorons et al. 2017; Woorons et al. 2019). This lower SpO_2_ may be associated with muscle (Woorons et al., 2007; Woorons et al. 2010; Woorons et al. 2017) and cerebral deoxygenation (Woorons et al., 2019).

A clear gap remains in the literature regarding whether the mechanisms underlying RSA improvements differ between traditional RSH and RSH-VHL, which induces additional stimuli such as hypercapnia, exaggerated intrathoracic pressure changes, and a potential apnoea reflex (Raberin et al., 2025). While the precise mechanisms have yet to be identified, recent meta-analysis suggests that RSH-VHL may enhance fatigue resistance beyond what is observed with RSN (Précart et al., 2025).

One may assume that VHL, due to the restricted breathing, has an impact on power output during a repeated-sprint exercise. However, Woorons et al. (2017) reported no significant difference in peak (PPO) and mean power output (MPO) between two cycling sessions of RSH-VHL and RSN. This is an important finding since RSH-VHL would allow participants to maintain the same training quality during a session as RSN.

Traditionally, as described in detail by Woorons et al. (2017), RSH-VHL is performed as follows: a normal exhalation prior to the sprint to reach functional residual capacity, breath-holding (apnoea) maintained throughout the sprint, followed by a second complete and rapid exhalation after the sprint to reach residual volume, before normal breathing resumes during inter-sprints recovery. Participants were unable to maintain apnoea for the full 6-s sprint. Indeed, they held their breath for 4–5 s before resorting to micro-breaths. Despite the shorter breath-holding duration, SpO_2_ dropped significantly from the second sprint onward in both the first and second sets during RSH-VHL (Woorons et al., 2017). This suggests that maintaining apnoea for the entire sprint duration is not necessary to elicit a hypoxic stimulus, and that an apnoea duration of 5 s is sufficient.

Female athletes are under-investigated (Cowley et al., 2021). Moreover, while sex differences in respiratory function are well known (Archiza et al., 2021; Dominelli and Molgat-Seon, 2022; Raberin et al., 2024), only one study has investigated RSH-VHL in females (Ait Ali Braham et al., 2024). This study demonstrated that female football players benefited from a 6-week RSH-VHL intervention to improve RSA during running. Training with end-expiratory breath-holding led to greater improvement in RSA performance compared to unrestricted breathing (Ait Ali Braham et al., 2024). End-of-sprint SpO_2_ was lower in the RSH-VHL group than in the RSN group (92.1% vs. 97.8%). In females, menstrual cycle could influence respiratory responses. At maximum intensities, hormonal influence on V̇E is minimal and physiological mechanisms appear to override hormonal mediation of ventilatory responses (Rattley et al., 2025). However, further study is needed to explore the acute effects of RSH-VHL in females, particularly in the context of cycling sprints, since no studies have yet been conducted on female participants.

In the present study, a modified VHL pattern was used (i.e., the continuous breathing protocol throughout each set (three times 6 min) with a fixed rhythm and a 5-s apnoea), to prolong the time spent at low lung volume and theoretically increase both systemic and local deoxygenation. We tested the hypothesis that RSH-VHL, with VHL performed continuously throughout each set, induces greater deoxygenation but similar power output to RSN.

## Methods

### Participants

Based on previous work showing correlations of 0.08–0.71 and large to very large effect sizes (Raberin et al., 2025), calculations using the lowest observed values indicated that eight participants would be sufficient to achieve 80% power at a 5% significance level. However, given the novelty of the VHL pattern, the exclusively female sample, and the aim to increase degrees of freedom, a more conservative approach was taken and thirteen participants were included. Thirteen healthy females voluntarily participated in the study (age: 25.5 ± 3.7 years; height: 164 ± 7 cm; weight: 57.6 ± 6.0 kg). The type of sport (team, individual and endurance sports) and the level (recreational or competitive) were heterogeneous. Most of the participants were sport sciences students. The inclusion criteria were as follows: being female, aged between 18 and 35 years, having no known illness, not being acclimatised to altitude, and being able to provide informed consent. All participants declared that they were in good health and had not spent one or more nights at an altitude above 2.500 m in the previous 3 months. Female participants were asked whether they were in a natural menstrual cycle or using hormonal contraception, and the date of their last menstrual period was recorded. Participants did not perform any additional exercise on the day of the sprint sessions. The study was approved by the Ethics Commission for the Protection of Human Beings (CER-VD 2023-01638) and conducted in accordance with the Declaration of Helsinski. All participants provided written informed consent after being fully briefed on the study’s nature, procedures, and potential risks.

### Experimental design

The study followed a randomised crossover design. The experimental protocol consisted of one familiarisation session followed by two test sessions.

### Familiarisation with the VHL technique

Each participant first completed a familiarisation session with the VHL technique prior to the first test session. This session lasted approximately 30 min, during which the participants performed 4 min of continuous VHL at rest in a standing position, and 4 min of continuous VHL while performing exercises, including squats and walking. Following familiarisation, participants underwent two sessions, separated by a minimum of 48 h and a maximum of 10 days.

**FIGURE 1:**
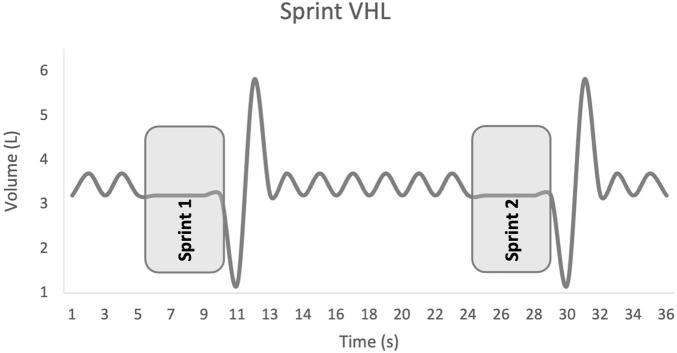
Sprint low lung volume voluntary hypoventilation (VHL). VHL was performed in other studies only during the sprints.

**FIGURE 2:**
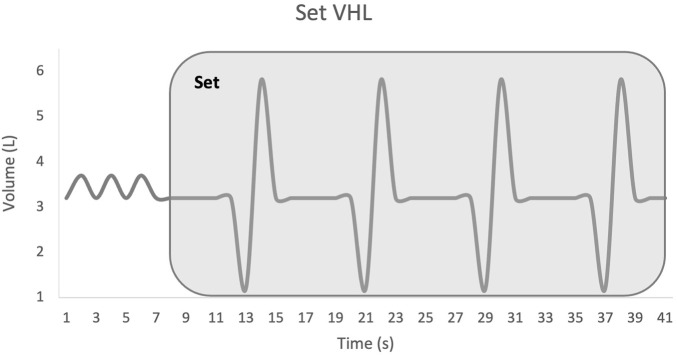
Set low lung volume voluntary hypoventilation (VHL). VHL was performed in this study during the entire set of sprints.

Participants performed the continuous VHL technique according to the following protocol: normal inspiration, normal expiration down to functional residual capacity, a 5-s apnoea, paced with a metronome, a rapid expiration of the expiratory reserve volume to reach residual volume, and again a normal inspiration and expiration, followed by repetition of the above cycle. Unlike previous studies where VHL was performed only during sprints (Figure 1), in the present study VHL was applied continuously 3 times for 6 min–including 2 min prior to each sprint set and during the 4 min of each RSH-VHL set (Figure 2). To prevent nasal breathing during apnoea, participants wore a nose clip throughout the continuous VHL phase.

### Repeated-sprint protocol

**FIGURE 3:**
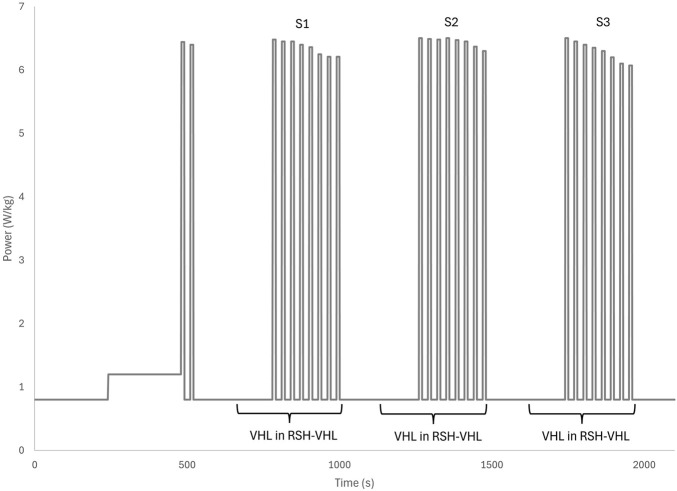
Sequence of continuous low lung volume voluntary hypoventilation (VHL) during repeated sprints in hypoxia induced by VHL (RSH-VHL). The three sets consist of eight all-out sprints of 10 s.

Repeated sprints were performed on a cycle ergometer (Excalibur, Lode, Groningen, Netherlands), beginning with an 8-min warm-up. The warm-up consisted of 4 min at 0.8 W/kg and 4 min at 1.2 W/kg, with cadence maintained between 60 and 90 rpm. After warming up, participants completed two sprints, followed by 4 min at 0.8 W/kg. During the RSH-VHL condition, the final 2 min of the 4 min pre-set period were performed using the continuous VHL technique (Figure 3).

The main protocol consisted of three sets of eight 10-s all-out sprints, each performed at a torque factor of 0.5 N/kg. Between sprints, participants had 20 s of recovery, during which they pedalled at 0.8 W/kg. A 10-s sprint with a 1:2 exercise-to-rest ratio was selected, as it has previously been shown to be optimal in RSH protocols (Raberin et al., 2022; Raberin et al., 2023). Recovery between sets lasted 4 min at 0.8 W/kg, with cadence maintained between 60 and 90 rpm. The second and third sets followed the same structure. After the final sprint set, participants completed a 2-min cooldown at 0.8 W/kg. The total session duration was 35 min.

### Measurements

#### Power output

PPO and MPO for each sprint were monitored using Version 10 of the Lode Ergometry Manager software (Lode, Groningen, Netherlands). PPO and MPO over the entire session were determined by averaging the values from the three sets.

#### Heart rate, stroke volume and cardiac output

Heart rate (HR), stroke volume (SV) and cardiac output (CO) were continuously monitored non-invasively using transthoracic cardioimpedance (Physioflow, Manatec Biomedical, Poissy, France), as previously described by Woorons et al. (2011). Data were exported at 1 Hz. The average values of each variable during the sprints, during the recovery periods between sprints for all three sets, and across the total session were calculated.

#### Blood lactate concentration

One minute after the final sprint of each set, a blood sample was taken from the ring finger to measure lactate concentration ([La]) using a BIOSEN C_Line analyser (EKF-diagnostic, Barleben, Germany).

#### Oxygen saturation and muscle oxygenation

SpO_2_ was measured continuously at the earlobe using the Avant ® 9600 device (NONIN, USA, MN) with a sampling frequency of 0.25 Hz. Data were exported using the nVision software. The average SpO_2_ of each set and the time spent below 96%, 94% and 92% SpO_2_ were calculated.

Muscle oxygenation in the right-leg vastus lateralis was assessed using near-infrared spectroscopy (NIRS) (PortaLite MKII, Artinis Medical Systems, Elst, Netherlands). The NIRS sensor was secured with double-sided tape and covered with an opaque layer to minimise light interference and enhance signal quality. The sensor’s position was marked with a permanent marker during the first session to ensure accurate repositioning in the second session. A differential pathlength factor (DPF) of four was used, as indicated by Woorons et al. (2017). Skinfold thickness (mean ± standard deviation) at the sensor site was measured (24.2 ± 8.9 mm).

NIRS data were collected at a sampling rate of 10 Hz and included the tissue saturation index (TSI), deoxyhaemoglobin concentration ([HHb]), oxyhaemoglobin concentration ([O_2_Hb]), and total haemoglobin concentration ([tHb]). Although [O_2_Hb] may be affected by rapid volume changes during sprints (Grassi et al., 2003), it was included in the analysis. [tHb], the sum of [O_2_Hb] and [HHb], represents the total blood volume in the muscle (Van Beekvelt et al., 2001). The minimum (min), maximum (max) and amplitude (Δ) for each variable were determined during the sets.

Non-locomotor muscle oxygen saturation (SmO_2_) was measured using a muscle NIRS system (MOXY Muscle Oxygen Monitor, Fortiori Design LLC, USA, MN) on the short head of the right biceps brachii. The MOXY device was securely fixed to prevent movement, and skinfold thickness (mean ± standard deviation) was also measured at the sensor site (6.3 ± 2.9 mm). The min during sprints, the max during recoveries, and the Δ SmO_2_ were calculated and used for analysis.

#### Perception: rating of perceived exertion, difficulty of breathing and pedalling

At the end of each set, participants reported their rating of perceived exertion (RPE) using the Borg scale (6-20). An analogue scale ranging from one to ten was also used to assess perceived difficulty of breathing and pedalling after each set.

### Statistical analysis

Statistical analyses were performed using Jamovi software. The normality of the data was assessed using Shapiro-Wilk test. Paired two-tailed t-tests were used to determine whether there were differences between RSH-VHL and RSN. If the data were normally distributed, a Student’s t-test was applied; otherwise, the Wilcoxon test was used. Statistical significance (p) was set at p < 0.05. All results are expressed as mean ± standard deviation (SD).

## Results

### Participants’ characteristics

Thirteen females participated in the study. One participant was excluded during the study due to sickness. All participants were physically active, with an average of 5.7 ± 3.8 h of exercise per week.

#### Power output

**FIGURE 4:**
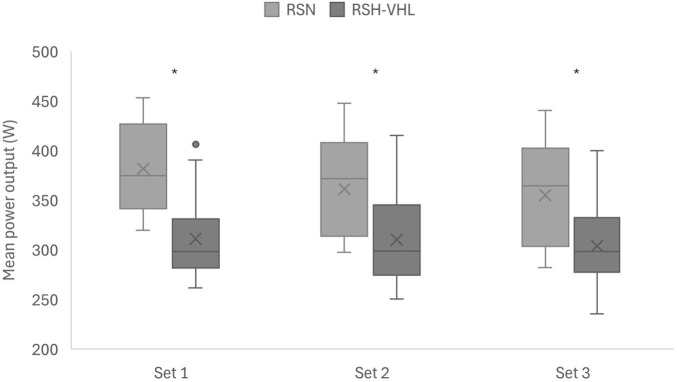
Mean power output for each set under both conditions: sprint repetitions in normoxia (RSN) and sprint repetitions in hypoxia induced by voluntary hypoventilation at low lung volume (RSH-VHL). * indicates that the difference between the two conditions is statistically significant (p < 0.001).

Training load indices were significantly lower during RSH-VHL compared with RSN from the first set. MPO in each set was significantly lower during RSH-VHL than during RSN (p < 0.001) (Figure 4). Over the entire session, MPO was also significantly lower during RSH-VHL compared to RSN (308 ± 45 vs. 366 ± 51 W; p < 0.001). PPO was significantly lower during RSH-VHL than RSN across all three sets: 409 ± 72 vs. 506 ± 80 W (p < 0.001) for the first set, 405 ± 76 vs. 467 ± 80 W (p < 0.001) for the second set, and 404 ± 74 vs. 460 ± 89 W (p < 0.001) for the third set. Overall, PPO across the session was significantly lower during RSH-VHL than RSN (406 ± 72 vs. 477 ± 82 W; p < 0.001).

#### Heart rate, stroke volume and cardiac output

HR was significantly lower during RSH-VHL than RSN for all sets (see haemodynamic variables in Table 1). SV was significantly lower during RSH-VHL last set compared to RSN. CO was significantly lower during RSH-VHL than RSN for the first two sets. No significant difference in CO was observed for the third set.

**TABLE 1:** Heart rate (HR), stroke volume (SV) and cardiac output (CO) for the three sets (S1, S2 and S3) under both conditions. Conditions were as follows: sprint repetitions in hypoxia induced by voluntary hypoventilation at low lung volume (RSH-VHL) or in normoxia (RSN). Statistics are expressed as T in a T-test and W in a Wilcoxon test. * indicates that the difference between the two conditions is statistically significant.

Haemodynamic variables		RSH-VHL	RSN	Statistics	p-value
HR (bpm)	S1	160 ± 14	168 ± 8	T = 2.68	0.023*
	S2	165 ± 11	172 ± 9	T = 2.96	0.014*
	S3	167 ± 11	172 ± 12	T = 2.91	0.017*
SV (mL)	S1	85.3 ± 17.6	92.6 ± 15.3	T = 1.58	0.144
	S2	90.0 ± 17.7	92.8 ± 14.4	T = 0.82	0.429
	S3	80.9 ± 17.1	92.2 ± 14.3	W = 57.00	0.032*
CO (L/min)	S1	13.9 ± 2.5	15.8 ± 2.4	T = 2.48	0.033*
	S2	14.4 ± 2.5	16.3 ± 2.4	T = 2.43	0.035*
	S3	14.5 ± 2.8	15.7 ± 2.8	T = 1.60	0.144

#### Blood lactate concentration

[La] was significantly lower during RSH-VHL than during RSN starting from the first set (p = 0.003 for the first set and p < 0.001 for the second and third sets) (see Table 2 for values).

**TABLE 2:** Concentration of lactate ([La]) for the three sets (S1, S2 and S3) under both conditions: sprint repetitions in hypoxia induced by voluntary hypoventilation at low lung volume (RSH-VHL) and sprint repetitions in normoxia (RSN). * indicates that the difference between the two conditions is statistically significant.

[La] (mmol/L)	RSH-VHL	RSN	T-test	p-value
S1	6.77 ± 2.88	9.88 ± 2.97	3.79	0.003*
S2	6.78 ± 2.88	10.60 ± 3.13	4.45	<0.001*
S3	6.42 ± 2.84	10.20 ± 3.08	4.84	<0.001*

#### Oxygen saturation and muscle oxygenation

**FIGURE 5:**
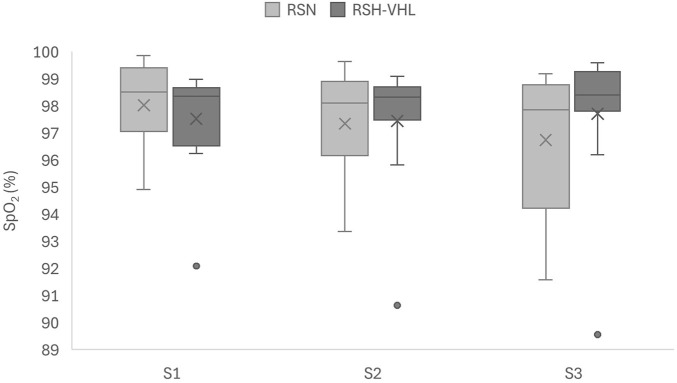
Pulse oxygen saturation (SpO_2_) for each set under both conditions. SpO_2_, expressed as a percentage (%), is represented for set 1 (S1), set 2 (S2) and set 3 (S3) depending on condition. Conditions were as follows: sprint repetitions in normoxia (RSN) and sprint repetitions in hypoxia induced by voluntary hypoventilation at low lung volume (RSH-VHL).

There was no significant difference in mean SpO_2_ between RSH-VHL and RSN (Figure 5) for the first (p = 0.243), second (p = 0.854) and third sets (p = 0.151). Likewise, time spent below SpO_2_ thresholds was not significantly different: <96% (p = 1.000), <94% (p = 0.944), and <92% (p = 1.000).

**FIGURE 6:**
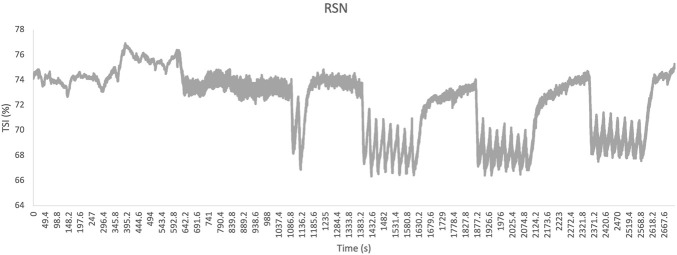
The raw signal of the tissue saturation index (TSI), expressed as a percentage (%), as a function of time in seconds (s) under normoxic sprint repetitions condition (RSN).

**FIGURE 7:**
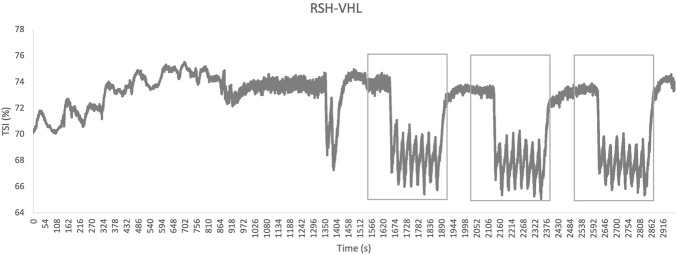
The raw signal of the tissue saturation index (TSI), expressed as a percentage (%), as a function of time in seconds (s) under the condition of repeated sprints in hypoxia induced by voluntary hypoventilation at low lung volume (RSH-VHL). The rectangles on the graph indicate the period with VHL: 2 min prior to the first sprint until the last sprint of each set (three times 6 min).

On the vastus lateralis, no significant differences were found in: min TSI (62.3% ± 4.3% vs. 61.5% ± 4.4%; p = 0.193), max TSI (69.7% ± 1.4% vs. 69.8% ± 2.6%; p = 1.543) and Δ TSI (7.5% ± 3.9% vs. 8.4% ± 3.5%; p = 0.375). It was not possible to identify the start of the VHL phase from the TSI signal (Figures 6, 7 for example).

The Δ[HHb] during the first set was significantly higher in RSH-VHL compared to RSN, but there was no significant differences in the second and third sets (see Table 3 for vastus lateralis oxygenation). The min [tHb] during sprints was significantly higher during RSH-VHL compared to RSN for: the first, second and third sets.

**TABLE 3:** Minimum (min), maximum (max) and amplitude (Δ) of the concentration of oxyhaemoglobin ([O_2_Hb]), deoxyhaemoglobin ([HHb]), and total haemoglobin ([tHb]) of the vastus lateralis for the three sets (S1, S2 and S3) under both conditions. Conditions were as follows: sprint repetitions in hypoxia induced by voluntary hypoventilation at low lung volume (RSH-VHL) or in normoxia (RSN). Statistics are expressed as T in a T-test and W in a Wilcoxon test. * indicates that the difference between the two conditions is statistically significant.

Vastus lateralis oxygenation	Phase	Set	RSH-VHL	RSN	Statistics	p-value
O_2_Hb (μm)	Min sprint	S1	−4.50 ± 4.01	−6.40 ± 4.02	T = −2.25	0.044*
		S2	−3.03 ± 4.75	−5.49 ± 3.38	T = −2.99	0.011*
		S3	−2.00 ± 4.88	−4.19 ± 2.87	T = −2.29	0.041*
	Max recovery	S1	3.82 ± 5.49	3.73 ± 4.31	T = −0.07	0.943
		S2	5.15 ± 5.34	4.16 ± 5.05	T = −0.63	0.542
		S3	−2.00 ± 4.88	−4.19 ± 2.87	T = −2.29	0.041*
	Δ	S1	8.31 ± 3.77	10.10 ± 3.93	T = 2.43	0.031*
		S2	7.95 ± 4.08	9.77 ± 6.28	W = 51.00	0.735
		S3	7.27 ± 3.88	7.09 ± 3.07	T = −0.26	0.796
HHb (μm)	Min recovery	S1	−1.41 ± 1.18	−2.02 ± 1.08	T = −2.62	0.022*
		S2	−1.10 ± 1.82	−1.12 ± 1.09	T = −0.06	0.953
		S3	−0.76 ± 1.87	−0.97 ± 1.20	T = −0.44	0.667
	Max sprint	S1	2.58 ± 1.62	3.35 ± 2.53	T = 1.81	0.096
		S2	2.94 ± 1.75	3.98 ± 3.14	W = 58.00	0.414
		S3	2.75 ± 1.92	2.71 ± 2.07	T = −0.08	0.939
	Δ	S1	−3.99 ± 2.22	−5.37 ± 2.94	−2.62	0.022
		S2	−4.03 ± 2.54	−5.10 ± 3.38	W = 38.00	0.635
		S3	−3.54 ± 2.32	−3.69 ± 2.65	−0.38	0.709
THb (μm)	Min sprint	S1	2.75 ± 1.92	2.71 ± 2.07	T = −0.08	0.939
		S2	7.18 ± 8.48	2.33 ± 4.33	T = −2.53	0.027*
		S3	8.84 ± 7.76	4.38 ± 3.20	T = −2.20	0.048*
	Max recovery	S1	10.50 ± 9.43	7.30 ± 4.97	T = −1.29	0.220
		S2	14.30 ± 8.92	10.10 ± 5.87	T = −2.05	0.062
		S3	14.80 ± 7.81	10.20 ± 4.95	T = −2.13	0.055
	Δ	S1	7.50 ± 3.52	8.93 ± 3.75	T = 1.73	0.109
		S2	7.15 ± 3.81	7.8 ± 3.38	T = 0.89	0.388
		S3	6.15 ± 3.44	6.08 ± 2.97	T = −0.12	0.910

In the biceps, no significant differences were observed in: min SmO_2_ (36.6% ± 10.0% vs. 34.2% ± 13.7%; p = 0.320) and max SmO_2_ (46.5% ± 8.6% vs. 48.5% ± 12.4%: p = 0.359). However, Δ SmO_2_ was significantly lower during RSH-VHL than RSN for all sets: 11.5% ± 5.5% vs. 16.0% ± 7.2% (p = 0.031) for the first set, 9.9% ± 3.4% vs. 14.7% ± 5.7% (p = 0.005) for the second set, and 8.6% ± 3.8% vs. 12.1% ± 3.9% (p = 0.008) for the third set.

#### Perception: rating of perceived exertion, difficulty of breathing and pedalling

RPE was not significantly different between the two conditions (17.2 ± 2.3 vs. 15.6 ± 1.6; p = 0.300). However, the perceived difficulty of leg effort was significantly lower during RSH-VHL compared to RSN from the second set onward (see Table 4). In contrast, the perceived difficulty of breathing was significantly higher during RSH-VHL than RSN across all three sets.

**TABLE 4:** Perception of difficulty for legs and breathing for the three sets (S1, S2 and S3) under both conditions. Conditions were as follows: sprint repetitions in hypoxia induced by voluntary hypoventilation at low lung volume (RSH-VHL) or in normoxia (RSN). Statistics are expressed as T in a T-test and W in a Wilcoxon test. * indicates that the difference between the two conditions is statistically significant.

Perception of difficulty		RSH-VHL	RSN	Statistics	p-value
Legs	S1	5.00 ± 2.35	5.62 ± 1.66	T = 1.425	0.180
	S2	5.85 ± 3.02	7.19 ± 1.47	T = 2.339	0.037*
	S3	6.00 ± 2.52	7.88 ± 1.50	T = 3.866	0.002*
Breathing	S1	8.19 ± 2.16	6.00 ± 0.91	W = 9.50	0.022*
	S2	8.69 ± 1.65	7.23 ± 1.52	W = 11.50	0.018*
	S3	8.96 ± 1.51	8.42 ± 1.32	T = 3.428	0.005*

## Discussion

The main finding of the present study is that VHL, performed continuously during each of the three repeated-sprint sets, did not reduce SpO_2_ or induce significant systemic or local hypoxia. Moreover, it led to a significantly lower power output.

MPO and PPO were significantly lower during RSH-VHL than during RSN. This contrasts with the findings of Woorons et al. (2017), where the training stimulus was similar between conditions. We expected power output to be similar in both conditions. In our view, the observed reduction in training quality during RSH-VHL is an important result and contradicts the outcomes commonly reported with RSH (Faiss et al., 2024; Millet et al., 2019). One of the objectives of RSH is to maintain maximal power during sprint efforts under hypoxic conditions, which differs from interval-training in hypoxia (Faiss et al., 2013; Faiss et al., 2024; Millet et al., 2019).

Regarding the cardiac response, HR was significantly lower during RSH-VHL than during RSN, as observed in previous studies: Ahn et al. (1989) involving apnoea during supramaximal exercise and Woorons et al. (2017). Ahn et al. (1989) and Woorons et al. (2017) attributed this lower HR to a mechanism involving peripheral arterial chemoreceptors activated by a drop in SpO_2_, and, thus hypoxia. In our case, the absence of systemic hypoxia suggests that the lower HR was more likely due to reduced cardiovascular demand associated with a lower exercise load during RSH-VHL. The difference in findings may be explained, on one hand, by the higher workload applied during RSN, and on the other hand, by a VHL implementation that may not have been optimal. An increase in CO during RSH-VHL was expected, as seen in moderate-intensity exercise (Woorons et al., 2011), potentially due to intrathoracic pressure changes from VHL (Woorons et al., 2011). However, CO was lower during RSH-VHL in our study, likely reflecting the reduced exercise load in this condition compared to RSN.

Blood lactate accumulation reflects an increased reliance on glycolytic metabolism. Since hypoxia reduces O_2_ availability and limits oxidative pathways, it promotes a greater dependence on glycolytic energy production (Burtscher et al., 2022; Maldonado-Rodriguez et al., 2022). In the present study, however, [La] were lower during RSH-VHL compared to RSN. Given that the hypoxic stimulus was identical (indeed absent) in both conditions, this difference cannot be attributed to hypoxia *per se*. During RSN, lactate concentrations (10.23 ± 3.06 mmol · L^-1^ across the three sets) were consistent with values typically observed in maximal, short, repeated-sprint bouts in females, which are known to be slightly lower than those reported in males (Piperi et al., 2024). In contrast, the lower lactate values observed during RSH-VHL (6.66 ± 2.86 mmol · L^-1^) most likely reflect the reduced exercise intensity in this condition, as evidenced by the lower mean power output generated across the three sets.

Contrary to our expectations, and unlike previous studies that reported significant desaturation with VHL (Ait Ali Braham et al., 2024; Fornasier-Santos et al., 2018; Woorons et al., 2016; Woorons et al., 2017; Woorons et al., 2019), SpO_2_ levels were not significantly different between the two conditions. Our findings are more in line with those of Rosa et al. (2024), who also reported no significant drop in SpO_2_ during upper-body RSH-VHL exercise. Several factors may explain the absence of hypoxemia. Woorons et al. (2014) demonstrated that voluntary hypoventilation at high pulmonary volume did not cause hypoxemia at sea level in swimmers. In the present study, breath-holding during RSH-VHL was probably not performed at low lung volume due to the extended duration over which it was applied. VHL is demanding for participants and very difficult to monitor for coaches or researchers, particularly because the lung volume at which apnoea is maintained cannot be controlled. This represents a major limitation of the method. The VHL technique requires extensive learning and cooperation from participants, often necessitating continuous verbal guidance or encouragement from investigator.

Muscle oxygenation, assessed via NIRS, remained similar between RSH-VHL and RSN. This result appears logical since SpO_2_ was similar in both sessions. It contradicts earlier findings where VHL was associated with greater muscle deoxygenation (Woorons et al., 2007; Woorons et al., 2010; Woorons et al., 2017). In our study, min [HHb] and Δ[HHb] were significantly higher during RSH-VHL. This suggests that there was no increased muscle deoxygenation during RSH-VHL, as the drop in [HHb], with the min reached at the end of recovery, was significantly greater during RSN. This could be explained by the higher power output observed under unrestricted breathing conditions. The observed increase in min [tHb] for all three sets may reflect changes in blood volume at the probe level, influenced by blood flow, vessel recruitment and vasodilation (Van Beekvelt et al., 2001). Muscle [tHb] decreased during each sprint, reaching a min at the end of each sprint, as previously shown in other repeated-sprints studies (Faiss et al., 2013; Raberin et al., 2022; Smith and Billaut, 2010). Muscle perfusion likely decreased further during RSN due to the greater effort and higher power output. During maximal exercise on a bicycle, muscle blood flow may be reduced due to intramuscular vascular occlusion (Raberin et al., 2022). Blood perfusion is restricted by intramuscular pressure in the vastus lateralis due to vessel compression, resulting in reduced blood flow during the exercise phase (Horiuchi et al., 2022; McNeil et al., 2015; Raberin et al., 2022).

No enhanced deoxygenation in the biceps was observed during RSH-VHL compared to RSN. On the contrary, Δ SmO_2_ was significantly higher during RSN, likely due to the greater power output and increased upper-body engagement (i.e., participants pulling harder on the cycle ergometer handlebars).

Although participants reported significantly greater breathing difficulty during RSH-VHL compared to RSN in all three sets, this did not translate into significantly reduced oxygen saturation. In terms of perceived exertion, RPE was not significantly different between the two conditions. Typically, RPE is higher with VHL (Ait Ali Braham et al., 2024; Woorons et al., 2016). The similar RPE between conditions may reflect a trade-off: greater respiratory difficulty during RSH-VHL, but lower mechanical effort due to reduced power output. As discussed below, this response may be specific to females. The fact that all participants were female is an important feature of the present study. Recent findings showed that RSH intervention compared to RSN improves RSA test to the same extent in females as in males (Piperi et al., 2024) and that females can benefit from RSH-VHL intervention (Ait Ali Braham et al., 2024). However, sex-based differences have been reported in the pulmonary system during exercise, with females more frequently experiencing exercise-induced hypoxemia (Raberin et al., 2024) and a higher metabolic cost of breathing compared to males (Kipp et al., 1985). Females may also be more sensitive to dyspnoea, as they are more likely to report inspiratory difficulty, a sensation of unsatisfied inspiration, and shallow breathing at the end of incremental exercise (Cory et al., 2015). These factors could influence both the execution of VHL and the perceived difficulty during RSH-VHL, potentially contributing to the observed reduction in power output to a greater extent in females than in males.

During strenuous exercise or hypoxic/apnoeic conditions, the respiratory muscles generate afferent feedback to the central nervous system. Exercise-induced fatigue of the diaphragm and accessory inspiratory muscles activates group III/IV phrenic afferents, which provide potent feedback to brainstem and cortical structures involved in effort perception (Romer and Polkey, 2008). This afferent input can increase sympathetic vasoconstriction in limb muscles, i.e., respiratory muscle metaboreflex, reducing locomotor muscle perfusion and accelerating peripheral fatigue (Harms et al., 2000; Dempsey et al., 2006). Moreover, cortical regions such as the insula and anterior cingulate cortex integrate respiratory-related sensory inputs, contributing to sensations of dyspnoea, air hunger, and global fatigue (Gandevia, 2001). Together, these mechanisms illustrate how apnoea and demanding ventilatory pattern may modulate central motor drive, influencing both power output and the perception of fatigue.

There are some limitations to this study. Menstrual cycles were recorded but not controlled in the present study. In females, menstrual cycle could influence some physiological responses. However, at submaximal and maximum intensities as it is the case for repeated-sprint training, hormonal influence is minimal and physiological response to exercise appear to override hormonal mediation of ventilatory responses (Eston and Burke, 1984; Prado et al., 2021; Rattley et al., 2025). Cofounding variables like diet or exercise during the week of test sessions were not controlled. VHL is difficult to monitor, particularly regarding lung volume during apnoea. The innovative aspect of the present VHL was the use of a continuous breathing protocol (three times 6 min) with a fixed-rate and 5-s breath-holds during all-out sprint repetitions sets. This protocol proved both challenging for participants to perform and for researchers to control, especially in ensuring that apnoeas were performed at low lung volume. It is likely that apnoea was not consistently maintained at the targeted lung volume.

## Conclusion

The main finding of the present study is that VHL, performed continuously during each of the three repeated-sprint sets, did not reduce SpO_2_ nor induced significant systemic or local hypoxia, contrary to our hypothesis. Moreover, it led to a significantly lower power output. A likely explanation is that apnoea during continuous VHL was not consistently maintained at a low lung volume, highlighting the technical difficulty of both applying and monitoring this method. Additionally, the lower power output observed during RSH-VHL may have confounded direct comparisons with RSN. Nevertheless, due to the sex differences in the pulmonary system, RSH-VHL may be more efficient in females than in males. Further studies are needed to determine whether the reported results of continuous VHL are specific to females and whether the decrease in power output would be less pronounced in males. Future studies should ensure adequate sport-specific familiarization prior to testing and provide clear instruction on the distinction between breath-holding at different lung volumes, as these variations elicit distinct physiological responses that may influence the outcomes of VHL protocols. Moreover, while a facemask may exacerbate dyspnoea, continuous monitoring of lung volume with a flowmeter during the session may be useful to ensure proper implementation of the VHL technique.

## Data Availability

The original contributions presented in the study are included in the article/supplementary material, further inquiries can be directed to the corresponding author.
